# P005: Effect of improvement activity for decreasing catheter related bloodstream infection

**DOI:** 10.1186/2047-2994-2-S1-P5

**Published:** 2013-06-20

**Authors:** MN Kim, NH Cho, YG Song, JH Yoon

**Affiliations:** 1 Department of Infection Control, Gangnam Severance Hospital, Seoul, Korea, Democratic People's Republic Of; 2 Department of Infectious disease, Gangnam Severance Hospital, Seoul, Korea, Democratic People's Republic Of

## Introduction

Insertion of central venous catheter is essential procedure for treatment of critical patient, but if this procedure is increased, bloodstream infection (BSI) can be increased. But through fulfillment of practice guideline, Catheter related Bloodstream Infection (CRBSI) can be fully prevented, and many hospitals try to decrease BSI like “0” percent.

## Objectives

In Intensive care unit (ICU) of this hospital, during the year 2010, BSI was 5.8/1000 device day but, it was increased like 12.2/1000 device day in April 2011. Therefore we started improvement activity to decrease BSI.

## Methods

We carried out improvement activities for three times for the patients who were inserted with central venous catheter from April 2011 to October 2012. **First (2011.04-2011.07),** 1) Prevention protocol review about BSI and sharing the protocol with relevant department, 2) SMS feedback to medical team about checklists and results with Maximum sterile barrier precaution, 3) Weekly experts (ID physician and IC nurse) rounding to ICU and CCU, and checking condition of central venous catheter. **Second (2012.05),** 1) Continuing previous improvement activity, 2) Sharing the protocol for sampling method of blood culture with relevant department, 3) Writing the name who inserted central venous catheter at the insertion site. **Third (2012.08-2012.10)**, 1) Notifying above protocols periodically, 2) Changing antiseptic from betadine to 2% alcoholic chlorhexidine gluconate solution since July 2012 when the commercial product was available.

## Results

When we compared CRBSI from first period (2011.04~06) to third period (2012.08~10), CRBSI was decreased as 45% from 10.1/1000 device day to 5.6/1000 device day in ICU and as 50% from 6.0 to 3.0 in whole wards

## Conclusion

We performed three improvement activities for decreasing CRBSI, we can confirm the effects of those activities. For decreasing CRBSI, continuous improvement activities are needed than temporary activities, and monitoring system which can monitor insertion practice of central venous catheter is also needed. After this, if we develop more effective system which can monitor the process, we can make larger effect.

## Disclosure of interest

None declared

